# Decrease in *PSCA* expression caused by *Helicobacter pylori* infection may promote progression to severe gastritis

**DOI:** 10.18632/oncotarget.23278

**Published:** 2017-12-14

**Authors:** Osamu Toyoshima, Chizu Tanikawa, Ryuta Yamamoto, Hidenobu Watanabe, Hiroharu Yamashita, Kosuke Sakitani, Shuntaro Yoshida, Michiaki Kubo, Keitaro Matsuo, Hidemi Ito, Kazuhiko Koike, Yasuyuki Seto, Koichi Matsuda

**Affiliations:** ^1^ Gastroenterology, Toyoshima Endoscopy Clinic, Tokyo, Japan; ^2^ Laboratory of Molecular Medicine, Human Genome Center, Institute of Medical Science, The University of Tokyo, Tokyo, Japan; ^3^ Department of Gastrointestinal Surgery, Graduate School of Medicine, The University of Tokyo, Tokyo, Japan; ^4^ Pathology and Cytology Laboratories (PCL) Japan, Tokyo, Japan; ^5^ Department of Gastroenterology, Graduate School of Medicine, The University of Tokyo, Tokyo, Japan; ^6^ Center for Integrative Medical Sciences, RIKEN, Yokohama, Japan; ^7^ Division of Molecular and Clinical Epidemiology, Aichi Cancer Center Research Institute, Nagoya, Japan; ^8^ Laboratory of Clinical Genome Sequencing, Department of Computational Biology and Medical Sciences, Graduate School of Frontier Sciences, The University of Tokyo, Tokyo, Japan

**Keywords:** genetic polymorphism, gene regulation, H. pylori infection, gastric cancer, Gastritis

## Abstract

SNP rs2294008 in *Prostate Stem Cell Antigen* (*PSCA*) and decreased *PSCA* expression are associated with gastric cancer. The objective of this study is to investigate the role of rs2294008 and *PSCA* expression in the gastritis-gastric cancer carcinogenic pathway. We conducted a case-control association study of *H. pylori*-infected gastritis and gastric cancer. rs2294008 was associated with the progression to chronic active gastritis (*P =* 9.4 × 10^–5^; odds ratio = 3.88, TT + TC vs CC genotype), but not with *H. pylori* infection *per se* nor with the progression from active gastritis to gastric cancer. We also assessed the association of rs2294008 with *PSCA* mRNA expression in the gastric mucosa at various disease stages and found that rs2294008 was associated with *PSCA* expression (*P =* 1.3 × 10^–12^). *H. pylori* infection (*P =* 5.1 × 10^–8^) and eradication therapy (*P* < 1 × 10^–11^) resulted in the reduced and increased *PSCA* expression, respectively, indicating negative regulation of *PSCA* expression by *H. pylori* infection. *PSCA* expression was decreased in severe gastritis compared with mild gastritis only among T allele carriers. Our findings revealed the regulation of *PSCA* expression by host genetic variation and bacterial infection might contribute to gastritis progression after *H. pylori* infection.

## INTRODUCTION

*Helicobacter pylori* colonizes the human gastric mucosa and has currently infected more than half of the entire human population [1]. The bacterium has been estimated to be the cause of approximately 90% of non-cardiac gastric cancers [2]. In addition to *H. pylori* virulence, host genetic backgrounds and other environmental factors also exhibit complex interactions in gastric cancer development. Gastric cancer develops through a multiple step process known as the gastritis-gastric cancer carcinogenic pathway that is triggered by *H. pylori* infection. Severe gastritis has been proven to be a precursor condition [1, 3–9]. In addition, *H. pylori* infection causes various diseases such as gastroduodenal ulcers and MALT lymphoma. Previous genome-wide association studies have revealed the association of SNP rs2294008 in *Prostate Stem Cell Antigen* (*PSCA*) with two *H. pylori* related diseases, gastric cancer and duodenal ulcer [10, 11]. The C allele of rs2294008 increases duodenal ulcer risk and the T allele increases gastric cancer and chronic atrophic gastritis risk [12], while rs2294008 was not associated with *H. pylori* susceptibility [13], suggesting the important role of this variation in clinical outcomes after *H. pylori* infection. rs2294008 was associated with *PSCA* mRNA expression in normal gastric tissues and gastric cancer. Although the gastric cancer-risk T allele is associated with a higher expression of *PSCA* mRNA, *PSCA* expression in gastric cancer tissues decreases. Therefore, the role of PSCA in gastric carcinogenesis remains largely unknown [10, 14]. Here, we investigated rs2294008 and *PSCA* expression using germline DNA and gastric mucosal tissues at different disease stages and revealed novel roles of host genetic factor and bacterial infection in the disease pathogenesis.

## RESULTS

### Study participants

The flowchart of patient enrollment was shown in Supplementary Figure 1. A total of 280 subjects with *H. pylori* infection and 28 *H. pylori*-negative controls were enrolled in this study [15]. Among 280 *H. pylori*-infected patients, 133 received the second evaluation after successful *H. pylori* eradication. Genotyping results of 509 *H. pylori*-negative controls and 2,329 gastric cancer patients analyzed in our previous study [11] were used in this study. The characteristics of the participants are summarized in Table 1 and Supplementary Table 1.

**Table 1:** Characteristics of study participants

	*H. pylori*-negative Controls	*H. pylori*-infection	*P* value^a^	*H. pylori*-negative Controls	Gastric cancer
Organizations	Toyoshima Endoscopy Clinic			Aichi Cancer Center	BioBank Japan
Samples	Blood DNA + gastric mucosal tissues			Blood DNA	
*n*	28	280		509	2,329
Age mean	50.2 +- 13.7	49.7 +- 11.9	0.93	42.0 +- 15.6	64.9 +- 9.1
Sex female %	46.4	49.6	0.75	52.7	22.2
Body mass index kg/m^2^	22.8 +- 2.67	22.3 +- 3.07	0.29		
Past malignancy except gastric cancer	1	18	1.0		
Family history of gastric cancer	6	47	0.60		
Drinking	9	71	0.43		
Smoking	5	20	0.063		
Gastric ulcer	1	28	0.44		
Duodenal ulcer	1	35	0.27		
Severe gastritis %	0	60.4	1.7 × 10^-9^		

509 *H. pylori*-negative controls and 2,329 gastric cancer cases were analyzed in our previous study (Tanikawa et al. 2012 Nature Genetics) ^11^. ^a^*P* values were calculated using Mann-Whitney *U* test, chi-square test, or Fisher’s exact test.

### The role of the SNP rs2294008 in the gastritis-gastric cancer carcinogenic pathway

**Figure 1:**
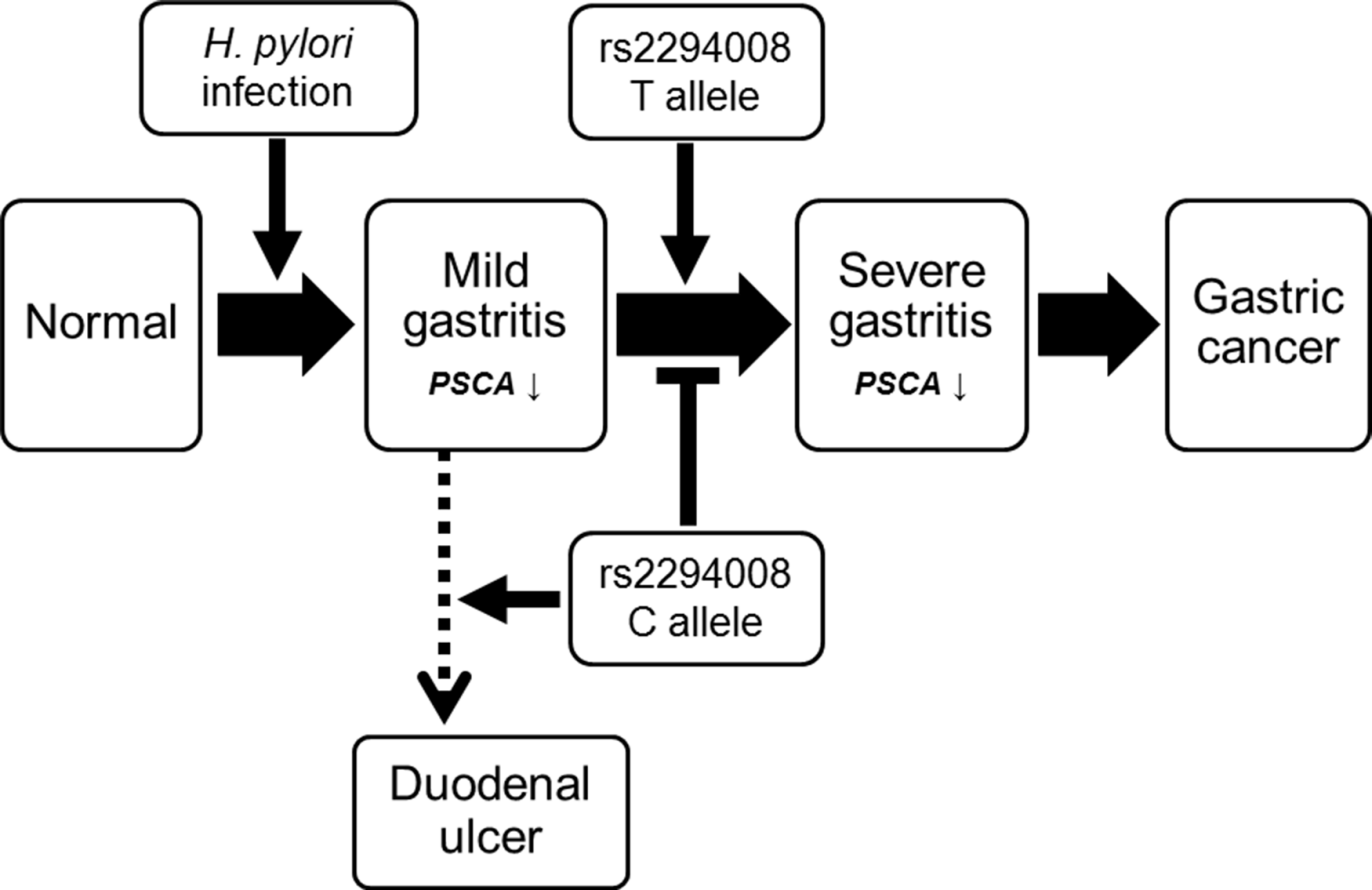
Scheme of SNP rs2294008 contribution to the diseases related with *H. pylori* infection including gastritis, gastric cancer, and duodenal ulcer

To investigate the role of the *PSCA* variation in gastric cancer and precancerous conditions, we genotyped rs2294008 using DNA from patients at different disease stages (Table 2). As previously reported [10, 12, 13, 16], the T allele of rs2294008 was significantly associated with gastric cancer risk when we used *H. pylori*-negative individuals as a control (*P* = 4.5 × 10^−6^; OR = 1.37). Then, we examined the impact of rs2294008 on the gastritis-gastric cancer carcinogenic pathway. There was no significant difference in the allelic frequency between *H. pylori*-carriers and *H. pylori*-negative controls (*P* = 0.15; OR = 1.14), indicating that rs2294008 is not associated with susceptibility to *H. pylori* infection. We then examined the impact of rs2294008 on the severity of gastritis as evaluated by histological neutrophil activity according to the updated Sydney system [17]. The individuals with *H. pylori* infection were divided into two categories, mild and severe gastritis [5], and rs2294008 was significantly associated with severe gastritis when we used mild gastritis or *H. pylori*-negative controls as references (*P* = 1.0 × 10^−3^ and 1.1 × 10^−3^ with OR of 1.93 and 1.52, respectively). However, rs2294008 was not associated with gastric cancer development from severe gastritis (*P* = 0.37; OR = 0.90). Similar tendencies were observed when gastritis was assessed by an endoscopic or a serological evaluation (Supplementary Table 2). These results indicated that rs2294008 was associated with the progression to severe gastritis after *H. pylori*-infection, but not with gastric cancer development from severe gastritis (Figure 1).

**Table 2:** Association of rs2294008 with gastritis-gastric cancer sequence

Cases	Controls	Cases			Controls			T vs C		TT + TC vs CC	
		CC	CT	TT	CC	CT	TT	*P*-value^a^	OR^b^ (95% CI)	*P*-value^c^	OR^d^ (95% CI)
Gastric cancer^e^	Hp- controls	201	1,087	1,041	73	275	189	4.5 × 10^–6^	1.37 (1.20–1.57)	4.2 × 10^–4^	1.67 (1.25–2.22)
Hp+	Hp- controls	41	120	119	73	275	189	0.15	1.14 (0.92–1.41)	0.68	0.92 (0.61–1.39)
Severe gastritis, Hp+	Mild gastritis, Hp+	13	74	81	27	45	38	1.0 × 10^–3^	1.93 (1.36–2.76)	9.4 × 10^–5^	3.88 (1.90–7.92)
Severe gastritis, Hp+	Hp- controls	13	74	81	73	275	189	1.1 × 10^–3^	1.52 (1.17–1.98)	0.043	1.88 (1.01–3.48)
Gastric cancer^e^	Severe gastritis, Hp+	201	1,087	1,041	13	74	81	0.37	0.90 (0.71–1.15)	0.69	0.89 (0.50–1.59)

Hp+: *H. pylori* positive, Hp-: *H. pylori* negative. ^a^*P* values are calculated by two-sided Cochran-Armitage test. ^b^ORs are calculated by considering the C allele as a reference. ^c^*P* values are calculated by chi-square test. ^d^ORs were calculated by considering the CC genotype as a reference. ^e^Data from the previous study (Tanikawa et al. 2012 Nature Genetics) ^11^.

The proportion of patients with severe gastritis was 32.5% among *H. pylori* carriers with the CC genotype, but it was as high as 62.2% and 68.1% among those with CT and TT genotypes, respectively. The comparison between the T carriers (TT or TC) and the CC genotype showed more remarkable associations, *P* = 9.4 × 10^−5^ and OR = 3.88. These results indicated that individuals with the TT or the TC genotype are susceptible to the progression to severe gastritis after *H. pylori*-infection and consequently have a high risk for gastric cancer.

### The association of the *PSCA* expression with SNP rs2294008, *H. pylori* infection, and the severity of gastritis

**Figure 2:**
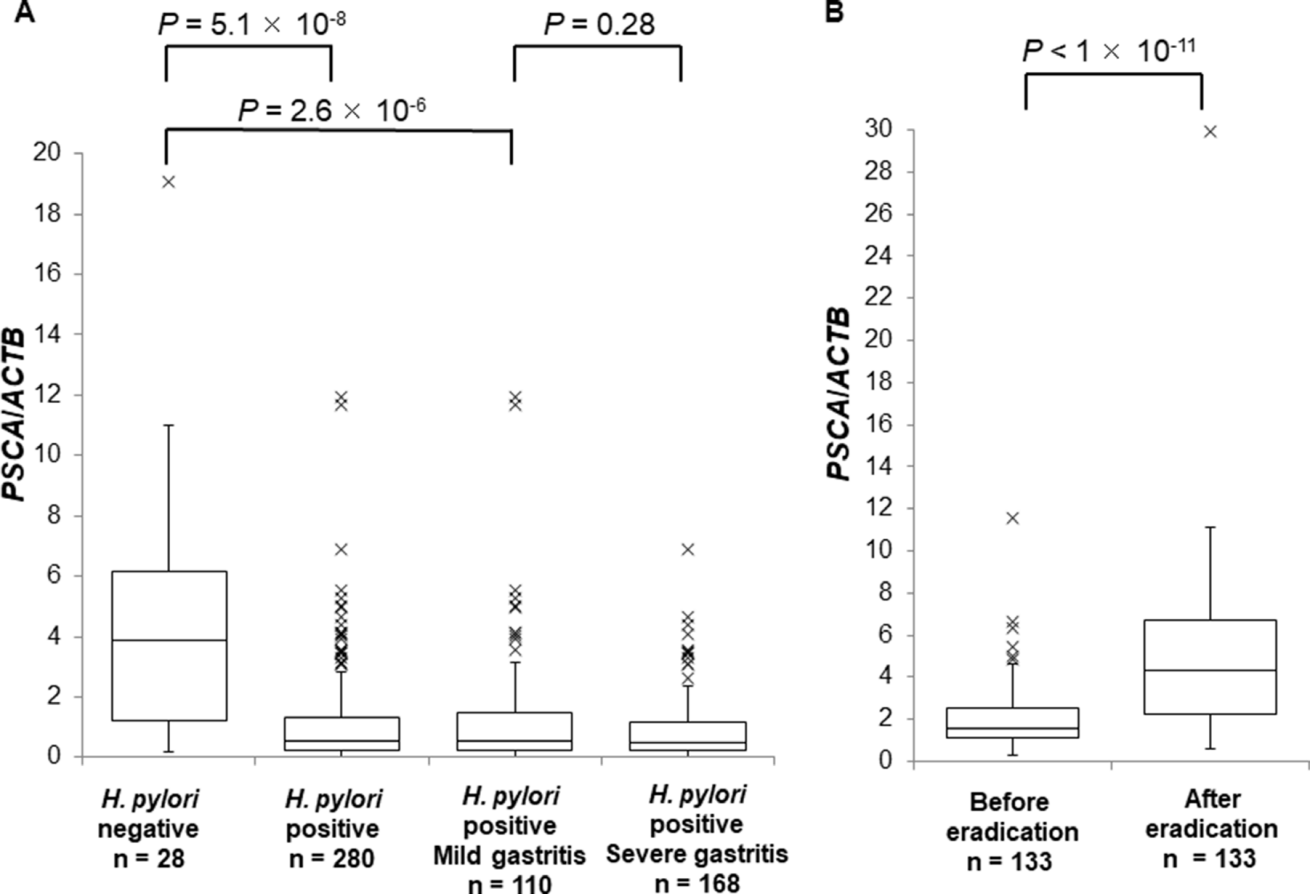
The suppression of *PSCA* expression by *H. pylori* infection Box-plots with medians for PSCA expression normalized to ACTB in biopsy samples of gastric mucosa. (**A**) *PSCA* mRNA in *H. pylori*-negative controls (*n* = 28), in *H. pylori*-infected patients (*n* = 280), in *H. pylori*-infected mild gastritis patients (*n* = 110), or in *H. pylori*-infected severe gastritis patients (*n* = 168). The *P* values were calculated by a Mann-Whitney *U* test. (**B**) The comparison of the expression levels of *PSCA* in each individual patient (*n* = 133) before and after *H. pylori* eradication. The *P* value was calculated by a Wilcoxon signed rank test.

**Figure 3:**
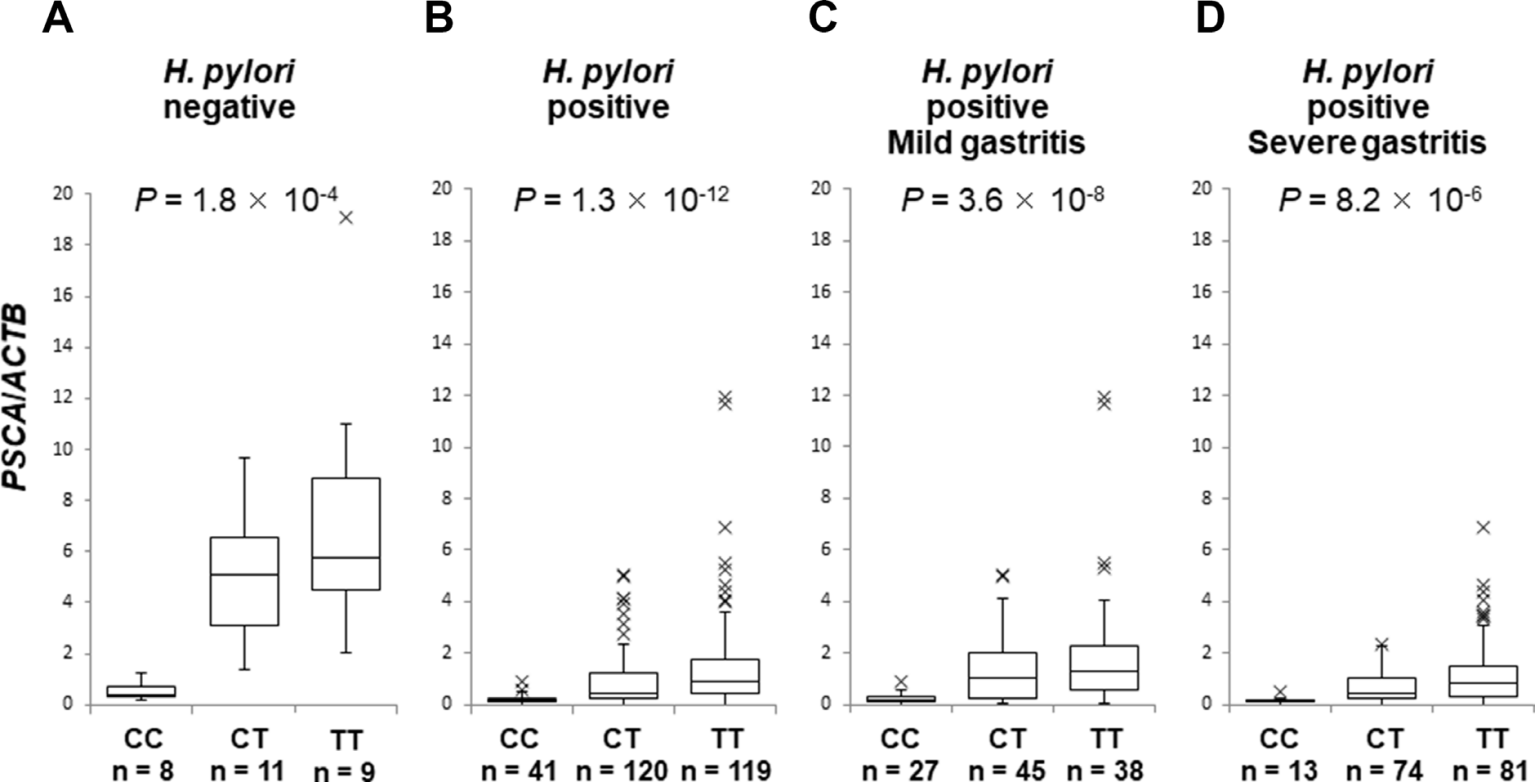
The association of SNP rs2294008 with *PSCA* expression Box-plots with medians for *PSCA* expression normalized to *ACTB* in biopsy samples of gastric mucosa. The *P* values were calculated by a Kruskal-Wallis test. (**A**) *PSCA* mRNA in *H. pylori*-negative controls (*n* = 28), (**B**) in *H. pylori*-infected patients (*n* = 280), (**C**) in *H. pylori*-infected mild gastritis patients (*n* = 110), (**D**) and in *H. pylori*-infected severe gastritis patients (*n* = 168).

Next, we evaluated *PSCA* mRNA in the background gastric mucosa of the incisura angularis at different disease stages. *PSCA* expression in *H. pylori*-infected patients (*n* = 280) was significantly lower than that in *H. pylori*-negative controls (*n* = 28) (Figure 2A, *P* = 5.1 × 10^−8^), but *PSCA* expression was not associated with gastritis stage (Figure 2A, *P* = 0.28). Moreover, *PSCA* expression increased after *H. pylori* eradication in 133 paired samples (Figure 2B, *P* < 1 × 10^−11^). These results clearly demonstrated the negative regulation of *PSCA* expression by *H. pylori* infection. Then, we analyzed the correlation of rs2294008 with *PSCA* expression. rs2294008 was significantly associated with *PSCA* expression at all disease stages (Figure 3A–3D, Supplementary Figure 2A–3D, *P* = 1.8 × 10^−4^ in *H. pylori*-negative, 1.3 × 10^−12^ in *H. pylori*-infected, 3.6 × 10^−8^ in mild gastritis, and 8.2 × 10^−6^ in severe gastritis). Consistent with our results, Sung *et al.* [14] reported that *PSCA* expression was associated with rs2294008 in gastric cancer tissues and non-tumor mucosa from gastric cancer patients. These results indicated that the T allele is associated with higher *PSCA* expression regardless of *H. pylori* infection status or disease stage.

**Figure 4:**
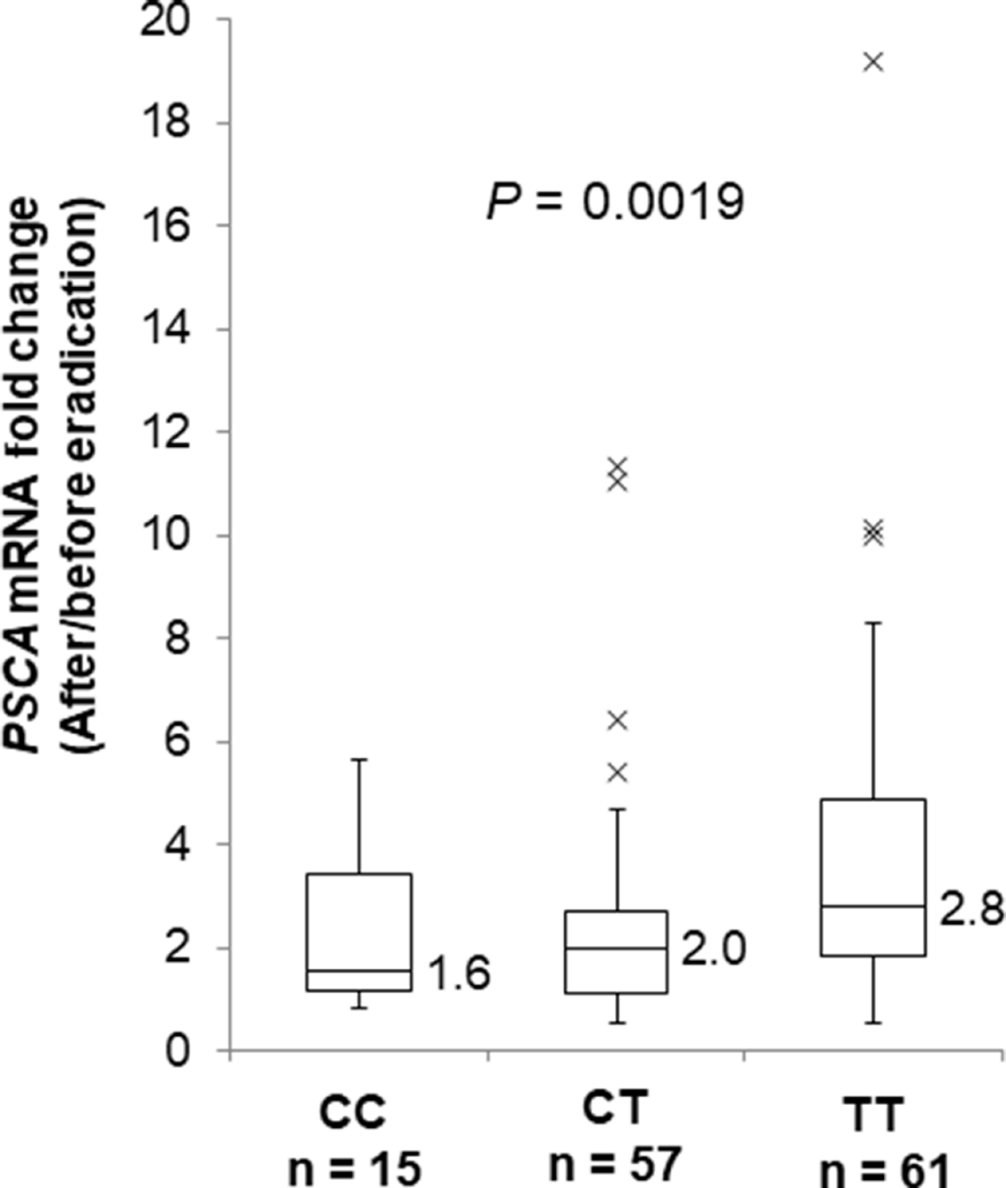
Fold *PSCA* mRNA induction by *H. pylori* eradication Box-plots with medians fold induction of *PSCA* expression normalized to *ACTB*. The fold induction was defined as *PSCA* expression after eradication divided by *PSCA* expression before eradication using paired samples. *PSCA* mRNA levels in paired samples before and after *H. pylori* eradication are quantified by the qRT-PCR analysis (CC; *n* = 15, CT; *n* = 57, TT; *n* = 61). The *P* values were calculated by a Kruskal-Wallis test.

In all three genotypes, *PSCA* expression was decreased in the gastric mucosa by *H. pylori* infection (Supplementary Figure 3A, CC: *P* = 0.0023, CT: 4.4 × 10^−7^, TT: 8.0 × 10^−6^) and was increased after *H. pylori* eradication (Supplementary Figure 3B, CC: *P* = 0.0038, CT: 3.6 × 10^−7^, TT: 2.7 × 10^−11^). Interestingly, *H. pylori* infection caused remarkable reductions in *PSCA* expression among T allele carriers compared with the CC genotype (Supplementary Figure 3A). We also evaluated *PSCA* mRNA change by *H. pylori* eradication in gastric mucosa by using paired samples before and after eradication. As a result, the T allele was associated with increased fold mRNA induction by *H. pylori* eradication (Figure 4, *P* = 0.0019). In addition, *PSCA* expression in severe gastritis patients was lower than in mild gastritis patients only among T carriers, but not among the CC genotype (Supplementary Figure 3C, CC: *P* = 0.36, CT: 0.048, TT: 0.032). Similar results were observed when the patients were assessed by endoscopic or serological evaluations (Supplementary Figure 4A, 4B). Altogether, *H. pylori* infection exhibited a stronger impact on *PSCA* expression among T allele carriers in the development of *H. pylori*-induced gastritis. Our findings clearly demonstrated that the regulation of *PSCA* expression by the host genetic factor (i.e., *PSCA* polymorphism) and the environmental factor (i.e., *H. pylori* infection) would play important roles in the pathogenesis of *H. pylori* related diseases.

## DISCUSSION

PSCA is a 123-amino acid glycoprotein that is related to the Ly-6 family of cell-surface proteins and has a role in signal transcription and in the regulation of cell proliferation. An increased expression of PSCA was observed in prostatic, bladder, and pancreatic cancers, indicating its oncogenic roles in tumor development of these cancers. However, decreased *PSCA* expression was documented in gastric cancer, gallbladder cancer, and head-and-neck squamous cell carcinoma, representing the complexity of PSCA function and *PSCA* regulation in various tissues [10–13, 16, 18–22]. Fu *et al.* [23] reported that the T allele of rs2294008 was associated with higher *PSCA* expression in both bladder cancers and in normal tissues. Because PSCA is upregulated in bladder cancer tissue and T allele carriers are associated with higher *PSCA* expression, the T allele is associated with a higher bladder cancer risk. However, the role of PSCA in gastric cancer development is not well understood, since *PSCA* expression was suppressed in gastric cancer tissues while the risk T allele is associated with higher *PSCA* expression.

Here, we identified the novel roles of a host genetic factor and bacterial infection in the regulation of *PSCA* expression in *H. pylori* induced active gastritis. rs2294008 was associated with disease progression to severe gastritis after *H. pylori* infection, but not with the development of gastric cancer after severe gastritis. In addition, a subgroup analysis using SNP genotypes revealed that only T allele carriers showed decreased *PSCA* expression in the progression from mild gastritis to severe gastritis. Here, we found that the CC genotype is associated with a low risk for progression to severe gastritis, which is consistent with our previous report that demonstrated an increased risk for duodenal ulcers in CC genotypes (Figure 1) [11, 24].

The number of active gastritis samples (*n* = 168) were relatively small compared with those of gastric cancer patients (*n* = 2,329). The statistical power of our study is estimated to be 62%, when it is assumed that OR of rs2294008 for gastric cancer risk is 1.3. However, information about *H. pylori* infection status of gastric cancer patients was not available in our study, although nearly 90% of gastric cancer patients were considered to be infected with *H. pylori*. Therefore, to conclude the role of rs2294008 in the progression from active gastritis to gastric cancer, further analysis using case-control samples with positive *H. pylori* infection is necessary. Currently we are conducting a follow-up survey of active gastritis patients. We would like to evaluate the role of genetic and clinical parameters as prognostic biomarkers in the future study.

rs2294008 is located 26 bases upstream of the translation initiation codon and the T allele encodes another translation initiation codon for the *PSCA* gene, resulting in an additional nine amino acids at N-terminal portion of PSCA protein to change protein localization from the cytoplasm to the cell surface [10, 11]. Long form of cell surface PSCA protein encoded by T allele was shown to promote cell proliferation [22, 25], while short cytosolic PSCA proteins associated with the C allele are rapidly degraded by the ubiquitin proteasomal pathway [11]. Therefore, C allele of rs2294008 is associated with low PSCA expression at mRNA and protein level. *H. pylori* infection has a strong impact among T allele carriers, because *H. pylori* infection remarkably suppress *PSCA* expression which is highly expressed in normal gastric mucosal tissues without *H. pylori* infection. In contrast, subjects with the CC genotype are unlikely to be affected by *H. pylori* infection because they express PSCA mRNA and protein at very low level regardless of *H. pylori* infection. Interestingly, individuals with a nonsense SNP (rs138377917[A]) of PSCA that lack functional PSCA protein is associated with a low risk for gastric cancer [26]. These lines of evidence clearly demonstrate that genotypes related with loss of functional PSCA (C allele of rs2294008 and A allele of rs138377917) are associated with a reduced risk for gastric cancer.

There are some limitations in this study because only endoscopy-based Japanese patients were analyzed. In addition, we did not examine CagA of *H. pylori*, but almost all Japanese *H. pylori* have CagA proteins and 95% of them are the East-Asia-type [1].

Here we revealed how host genetic variation and bacterial infection regulate *PSCA* expression and progression to severe gastritis. Our findings elucidated the critical roles of *PSCA* expression and SNP in the gastritis-gastric cancer progression and their possible implications for risk prediction and personalized disease prevention.

## MATERIALS AND METHODS

### Study design and participants

This cohort study consisted of subjects who agreed to participate in this study and underwent esophagogastroduodenoscopy (EGD) at Toyoshima Endoscopy Clinic from December 2013 to April 2015. We could obtain a written informed consent from a total of 326 individuals. All EGDs were undertaken by experienced endoscopists. EGDs were performed either for screening, an evaluation of present symptoms, surveillance of previous esophagogastroduodenal disease, positive results of *H. pylori* infection, an abnormal serum pepsinogen level, or abnormal findings on the barium meal. The inclusion criteria were patients aged 20 years or more without a past history of gastric cancer, esophagogastric junction adenocarcinoma, surgical gastrectomy, or *H. pylori* eradication. The exclusion criteria were a diagnosis of gastric cancer or adenoma based on an EGD at the time of enrollment, autoimmune gastritis, severe concomitant illness, an unidentified status of *H. pylori*, or an agreement withdrawal. We diagnosed patients with histologically severe corpus-restricted atrophic gastritis as autoimmune gastritis [27].

We divided the participants into two groups, with or without current *H. pylori* infection. We defined participants with histological intestinal metaplasia or a serum pepsinogen I/II ratio <3.0 as infected with *H. pylori* in the past and excluded them from the *H. pylori*-negative control groups [28]. Participants infected with *H. pylori* received eradication therapy after informed consent was provided. After the confirmation of successful *H. pylori* eradication, we performed the second EGD 6 months to 2 years after the index EGD. We collected peripheral blood samples at enrollment. The remaining mucosal biopsy tissues from the gastric incisura angularis at the index and the second EGD were used in this study.

*H. pylori* negative controls (*n* = 509) and gastric cancer patients (*n* = 2,329) were recruited at Aichi Cancer Center and BioBank Japan, respectively [11].

This research project was approved by the institutional review board at each institute. All participants provided written informed consent as approved by the institutional review board.

### Assessment of *H. pylori* status

At enrollment, we determined current *H. pylori* infection by either a positive result of the ^13^C-urea breath test (UBIT, Otsuka, Tokushima, Japan), the stool antigen test, or a combination of the serum immunoglobulin G antibody test (E-plate, Eiken, Tokyo, Japan) and culture or pathology (hematoxylin and eosin staining). The ^13^C-urea breath test requires a 2-week cessation of maintenance therapy with acid suppressors. At least 8 weeks after the completion of eradication therapy, successful eradication was confirmed using a negative ^13^C-urea breath test or stool antigen test [7].

### Diagnosis of gastritis

We divided gastritis into two categories, mild and severe gastritis, by three methods: histology, endoscopy, and serology.

The histological estimation was conducted according to the updated Sydney system [17]. To grade the neutrophil activity, we used two biopsy specimens from the greater curvature of the corpus and the antrum obtained by the EGD. We topographically classified the gastritis into four categories (i.e., no gastritis, antrum-predominant gastritis, pangastritis, and corpus-predominant gastritis). No gastritis and antrum-predominant gastritis were defined as mild gastritis and pangastritis and corpus-predominant gastritis were defined as severe gastritis. The histological diagnosis was performed by an expert gastrointestinal pathologist independently of the endoscopists. Chronic active gastritis identified by the distribution of neutrophil activity represents persistent active inflammation that is associated with tissue damage. Chronic active gastritis has been proven to be strongly associated with gastric cancer (corpus-predominant gastritis; relative risk [RR]: 34.5 [95% CI: 7.1−166.7], pangastritis; 15.6 [6.5−36.8], antrum-predominant gastritis as the reference group) [5, 29].

Endoscopic gastritis was assessed by atrophic-border identification by the different color and height of the mucosa according to Kimura-Takemoto classification [30]. In closed-type atrophic gastritis, the atrophic-border lies between the antrum and the lesser curvature of the gastric body and is defined as mild gastritis. In open-type gastritis, the border lies beyond the cardia and reaches the greater curvature of the gastric body and is defined as severe gastritis. The endoscopic-atrophic-border represents the histological border of atrophy and the range of atrophy spreads with the progression of gastritis [31]. Endoscopic gastritis has been demonstrated as a risk factor for gastric cancer (severe gastritis; RR: 4.9 [95% CI: 2.8–19.2], none or mild gastritis as the reference group) [5].

We used serum levels of pepsinogen I and II for the serological evaluation and defined a pepsinogen I/II ratio <3.0 as severe gastritis [8, 32]. Serum levels of pepsinogen I and II were measured using a commercially available kit (Pepsinogen CLEIA; Fuji Rebio Ltd., Tokyo, Japan). We excluded patients receiving proton pump inhibitors from the serological evaluation. The pepsinogen I/II ratio has been shown to be associated with the risk for gastric cancer (OR: 2.78−10.92) [8, 33].

### *H. pylori* eradication

The *H. pylori* eradication first-line regimen included clarithromycin 400 mg, amoxicillin 1500 mg, and a daily proton pump inhibitor for 7 days. In patients with infection of clarithromycin-resistant *H. pylori* or those with allergies to clarithromycin, the second-line regimen included metronidazole 500 mg, amoxicillin 1500 mg, and a daily proton pump inhibitor for 7 days.

### SNP Genotyping

DNA was isolated from peripheral blood leukocytes by using QIAamp DNA mini Kits (Qiagen, Valencia, CA, USA) according to the manufacturer’s instructions. The samples were genotyped by the Invader assay system (Third Wave Technologies Madison, WI) using the purified DNA form peripheral blood. Aliquot of DNA from patients with active or mild gastritis was randomly assigned to a 96 well plate and subjected to SNP analysis. The genotype results of healthy controls and gastric cancer patients analyzed in the previous study was used in this study [11].

### qRT-PCR analysis

The RNA was extracted from the fresh mucosa of the gastric angulus as background by EGD biopsy. Total RNA were isolated from human tissues using AllPrep DNA/RNA/miRNA Universal Kits (Qiagen, Valencia, CA, USA) according to the manufacturer’s instructions. RNA was treated with DNase during purification. Quality and quantity of RNA were evaluated by using NanoDrop (Thermo Scientific). Total RNA with > 1.9 of OD 260/280 ratio were used for further analysis. Complementary DNAs were synthesized from 500 ng of total RNA using Super Script III reverse transcriptase (Invitrogen). Quantitative real-time PCR (qRT-PCR) was conducted using SYBR Green Master Mix with a Light Cycler 480 (Roche, Basel, Switzerland). The expression of the *beta*-*actin* (*ACTB*) gene was used for normalization. A serial dilution of plasmid containing PCR fragment and distilled water (no template control) were used to calculate copy number of *ACTB* and *PSCA*. Primers used for qRT-PCR were as follows; PSCA_F:ctgctgtgctactcctgcaa, PSCA_R:ttgctgatgacggtcagg, ACTB_F:ccctggagaagagctacgag, ACTB_R:tgaaggtagtttcgtggatgc.

### Statistical analysis

The comparison of the demographic characteristics between the groups was performed using the Mann-Whitney *U*-test in continuous variables and the chi-square test or Fisher’s exact test in categorical variables. The association of rs2294008 with various diseases related with *H. pylori* infection was tested by the two-sided Cochran-Armitage trend test among the three genotypes and by the chi-square test between the CC genotype and T carriers. The odds ratios were calculated by considering the C allele or the CC genotype as reference. The comparison of the gene expression between the two groups was analyzed using the Mann-Whitney *U*-test and among the three groups using the Kruskal-Wallis test. The comparison of gene expression before and after *H. pylori* eradication was analyzed using the Wilcoxon signed rank test. Significance was indicated by a *P* value less than 0.05. Calculations were carried out using statistical software Ekuseru-Toukei 2015 (Social Survey Research Information Co., Ltd., Tokyo, Japan).

## SUPPLEMENTARY MATERIALS FIGURES AND TABLES



## Abbreviations

PSCA: Prostate Stem Cell Antigen
SNP: single nucleotide polymorphism
*H. pylori*: *Helicobacter pylori*
EGD: esophagogastroduodenoscopy
RR: relative risk
CI: confidence interval
OR: odds ratio
qRT-PCR: quantitative real-time PCR
*ACTB*: beta-actin
