# Supplementary material for: Decrease in PSCA expression caused by Helicobacter pylori infection may promote progression to severe gastritis
Source: Oncotarget. 2017 Dec 14;9(3):3936–45. doi: 10.18632/oncotarget.23278 (PMC5790512; doi:10.18632/oncotarget.23278)
Supplement: Supplementary file 1 [file oncotarget-09-3936-s001.pdf]

Decrease in *PSCA* expression caused by *Helicobacter pylori* infection may promote progression to severe gastritis

SUPPLEMENTARY MATERIALS

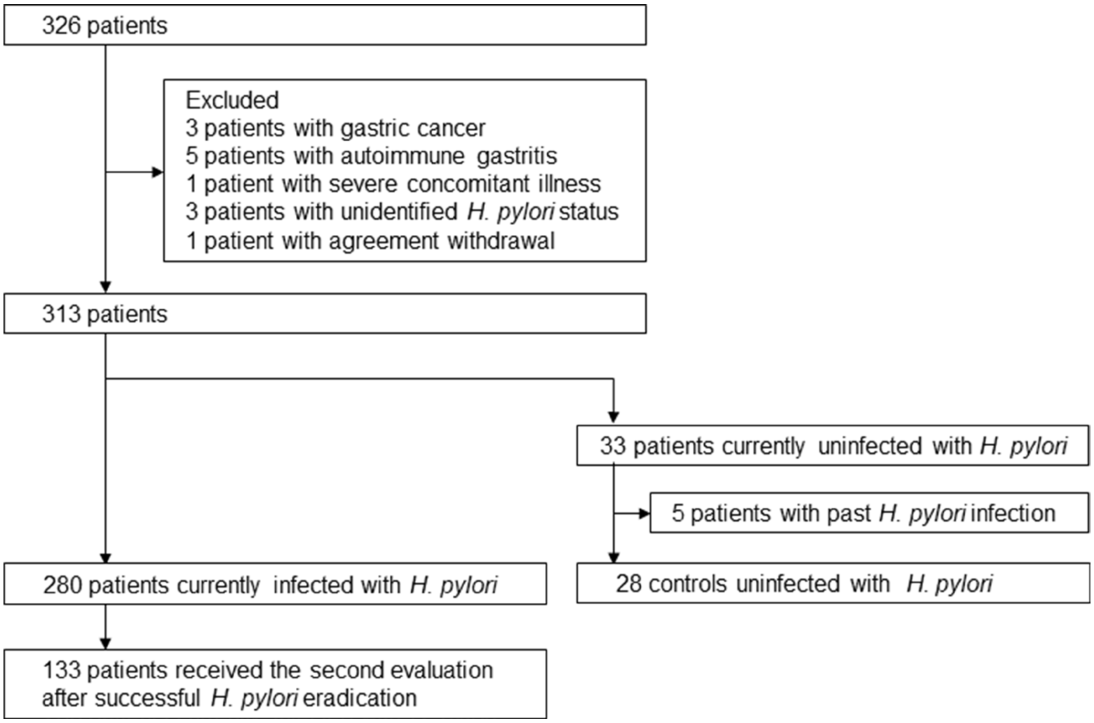

Supplementary Figure 1: Flowchart of inclusion and exclusion criteria of patients who underwent esophagogastroduodenoscopy.

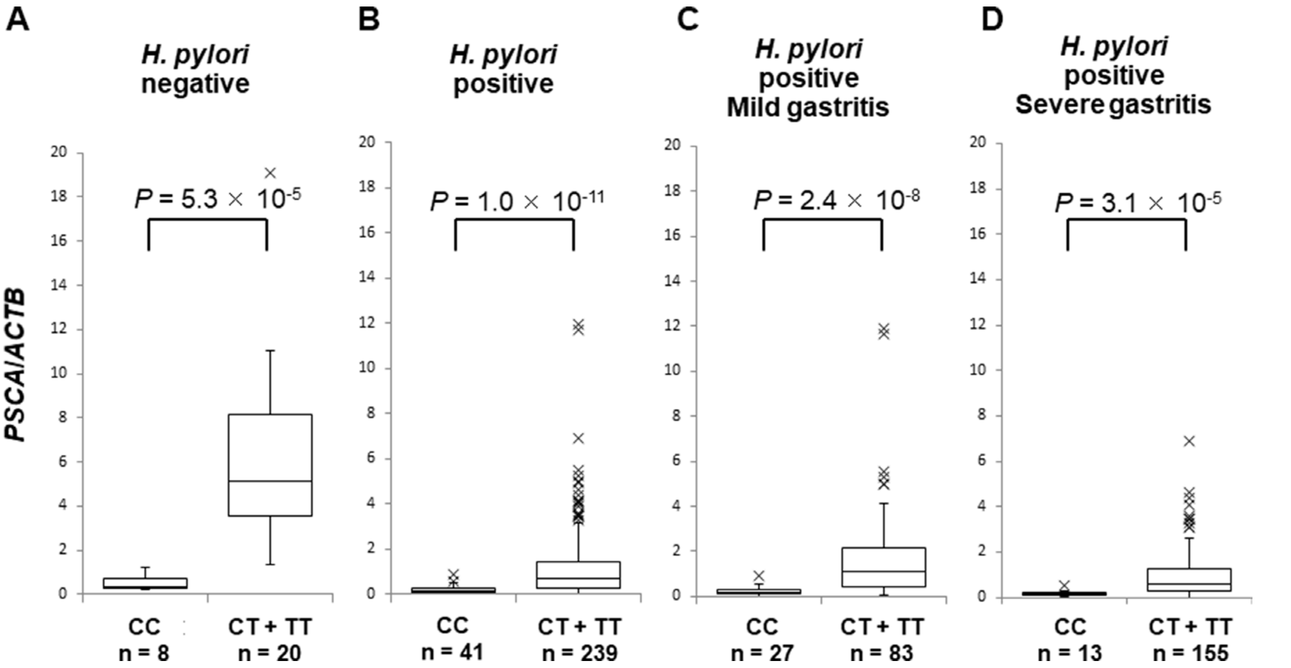

Supplementary Figure 2: The association of SNP rs2294008 with PSCA expression. Box-plots with medians for PSCA expression normalized to ACTB in biopsy samples of gastric mucosa. The P values were calculated by a Mann-Whitney *U*-test. (**A**) PSCA mRNA in *H. pylori*-negative controls (*n* = 28), (**B**) in H. pylori-infected patients (*n* = 280), (**C**) in *H. pylori*-infected mild gastritis patients (*n* = 110), (**D**) and in *H. pylori*-infected severe gastritis patients (*n* = 168).

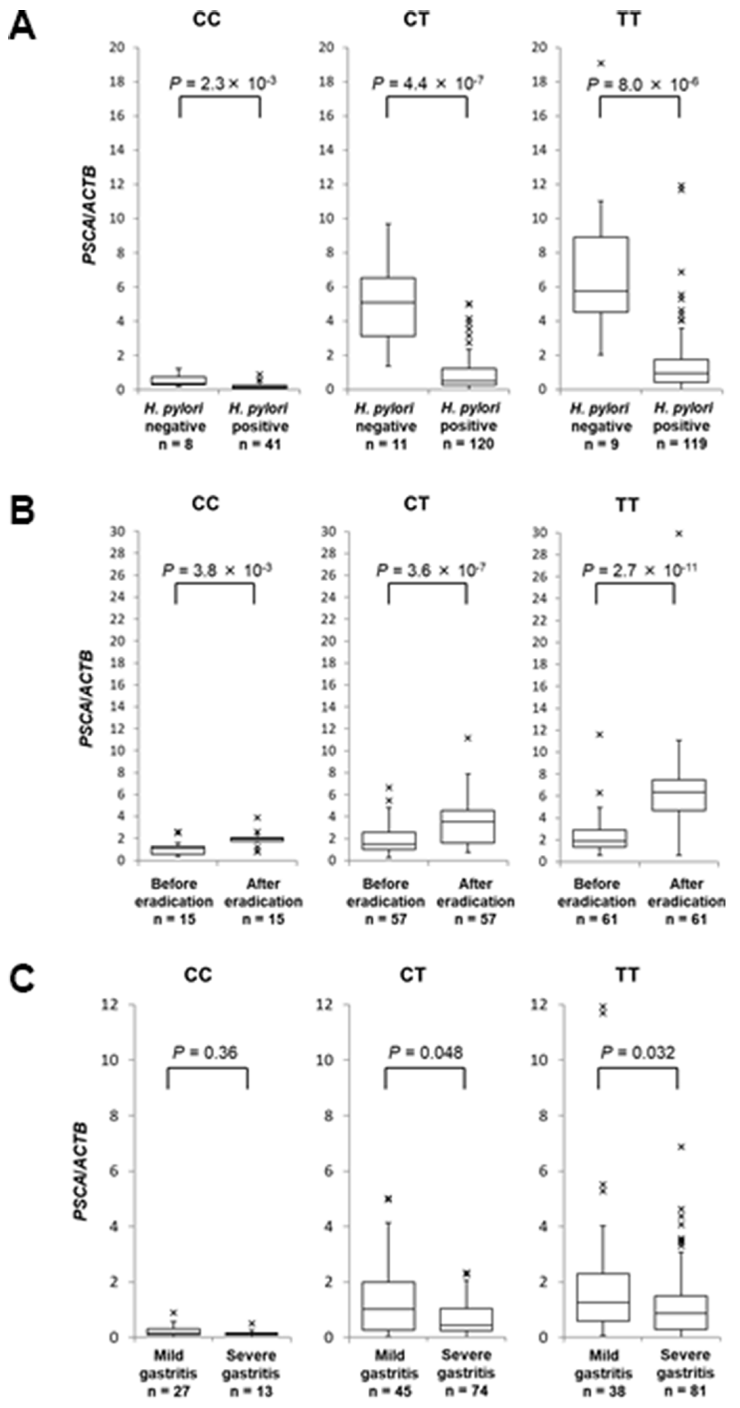

Supplementary Figure 3: The host-bacterial interaction in the regulation of PSCA expression. Box-plots with medians for PSCA expression normalized to ACTB in biopsy samples of gastric mucosa. The P values were calculated by a Mann-Whitney *U*-test. (**A**) The subgroup analysis of PSCA mRNA compared between *H. pylori*-negative controls (n = 28) and *H. pylori*-infected patients (n = 280). (**B**) The subgroup analysis of PSCA mRNA compared before and after *H. pylori* eradication (n = 133). (**C**) The subgroup analysis of PSCA mRNA compared between *H. pylori*-infected patients with mild gastritis (n = 110) and those with severe gastritis (n = 168).

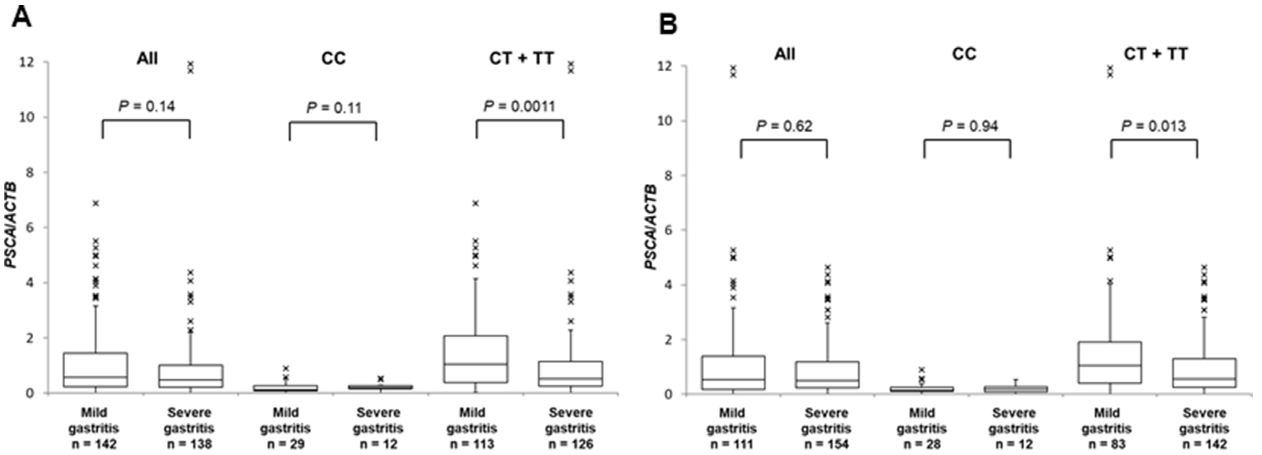

Supplementary Figure 4: The genotype-specific impact of *PSCA* expression in the development of severe gastritis. Box-plots with medians for PSCA expression normalized to ACTB in biopsy samples of gastric mucosa. The *P* values were calculated by a Mann-Whitney U-test. (**A**) By endoscopic evaluation, the subgroup analysis of PSCA mRNA compared between *H. pylori*-infected patients with mild gastritis (n = 142) and those with severe gastritis (n = 138). (**B**) By serological evaluation, the subgroup analysis of PSCA mRNA compared between *H. pylori*-infected patients with mild gastritis (n = 111) and those with severe gastritis (n = 154).

Supplementary Table 1: Proportion of gastritis

	H. pylori-negative controls	*H. pylori*-infected	*P* value[a]	Before/after[b]	*P* value[a]
Histological gastritis (%)			1.7×10^{-9}		6.8×10^{-31}
Mild	27 (100)	110 (40)		41/129	
Severe	0	168 (60)		88/0	
Endoscopic gastritis (%)			8.6×10^{-4}		0.013
Mild	24 (86)	142 (51)		52/72	
Severe	4 (14)	138 (49)		79/59	
Serological gastritis (%)			4.7×10^{-8}		5.3×10^{-19}
Mild	25 (100)	111 (42)		33/95	
Severe	0	154 (58)		70/8	

We analyzed 28 *H. pylori*-negative controls and 280 H. pylori-infected patients. [a]*P* values were calculated by chi-square test. Among 280 *H. pylori*-infected patients, 133 patients (129 by histology, 131 by endoscopy, and 103 by serology, respectively) were evaluated more than 6 months after *H. pylori* eradication[b].

Supplementary Table 2: Frequency of PSCA rs2294008 SNP in *H. pylori* infection-gastritis-gastric cancer sequence evaluated by endoscopy or serology

Cases	Controls	Cases			Controls			T vs C		TT + TC vs CC	
		CC	CT	TT	CC	CT	TT	*P*-value[a]	OR[b] (95% CI)	*P*-value[c]	OR[d] (95% CI)
SG, Endoscopy, Hp+	MG, Endoscopy, Hp+	12	60	66	29	60	53	0.013	1.62 (0.99–2.66)	5.5×10^{-3}	2.69 (1.31–5.53)
SG, Serology, Hp+	MG, Serology, Hp+	12	69	73	28	44	39	1.9×10^{-3}	1.82 (1.27–2.60)	9.2×10^{-5}	3.99 (1.92–8.27)
SG, Endoscopy, Hp+	Hp- controls	12	60	66	73	275	189	4.8×10^{-3}	1.47 (1.11–1.96)	0.12	1.65 (0.87–3.14)
SG, Serology, Hp+	Hp- controls	12	69	73	73	275	189	6.3×10^{-4}	1.59 (1.20–2.11)	0.090	1.73 (0.91–3.28)
Gastric cancer[e]	SG, Endoscopy, Hp+	201	1,087	1,041	12	60	66	0.53	0.93 (0.72–1.21)	0.98	1.01 (0.55–1.85)
Gastric cancer[e]	SG, Serology, Hp+	201	1,087	1,041	12	69	73	0.50	0.92 (0.72–1.18)	0.72	0.89 (0.49–1.64)

SG: severe gastritis, MG: mild gastritis. Hp+: *H. pylori* positive, Hp-: *H. pylori* negative. [a]*P* values are calculated by two-sided Cochran-Armitage test. [b]ORs are calculated by considering the C allele as a reference. [c]*P* values are calculated by chi-square test. [d]ORs were calculated by considering the CC genotype as a reference. [e]Data from the previous study (Tanikawa et al. 2012 Nature Genetics)[11].
